# Ubiquitin carboxyl-terminal hydrolase-L5 promotes TGFβ-1 signaling by de-ubiquitinating and stabilizing Smad2/Smad3 in pulmonary fibrosis

**DOI:** 10.1038/srep33116

**Published:** 2016-09-08

**Authors:** Ling Nan, Anastasia M. Jacko, Jiangning Tan, Dan Wang, Jing Zhao, Daniel J. Kass, Haichun Ma, Yutong Zhao

**Affiliations:** 1 Department of Anesthesia, First Hospital of Jilin University, Changchun, China; 2 Department of Medicine, Acute Lung Injury Center of Excellence, Department of Cell Biology, University of Pittsburgh, Pittsburgh, PA, United States

## Abstract

Transforming growth factor β-1 (TGFβ-1)-induced phosphorylation of transcription factors Smad2 and Smad3 plays a crucial role in the pathogenesis of idiopathic pulmonary fibrosis (IPF). However, the molecular regulation of Smad2/Smad3 proteins stability remains a mystery. Here, we show that ubiquitin carboxyl-terminal hydrolase-L5 (UCHL5 or UCH37) de-ubiquitinates both Smad2 and Smad3, up-regulates their stability, and promotes TGFβ-1-induced expression of profibrotic proteins, such as fibronectin (FN) and α-smooth muscle actin (α-SMA). Inhibition or down-regulation of UCHL5 reduced Smad2/Smad3 levels and TGFβ-1-induced the expression of FN and α-SMA in human lung fibroblast. We demonstrate that Smad2 and Smad3 ubiquitination was diminished by over-expression of UCHL5, while it was enhanced by inhibition or down-regulation of UCHL5. UCHL5 is highly expressed in IPF lungs. UCHL5, Smad2, and Smad3 levels were increased in bleomycin-injured lungs. Administration of UCHL5 inhibitor, b-AP15, reduced the expression of FN, type I collagen, Smad2/Smad3, and the deposition of collagen in lung tissues in a bleomycin-induced model of pulmonary fibrosis. Our studies provide a molecular mechanism by which UCHL5 mitigates TGFβ-1 signaling by stabilizing Smad2/Smad3. These data indicate that UCHL5 may contribute to the pathogenesis of IPF and may be a potential therapeutic target.

Idiopathic pulmonary fibrosis (IPF) is a chronic, progressive fibrotic lung disease characterized pathologically by excessive production and deposition of extracellular matrix (ECM). Abnormal re-epithelization and repair following an unknown injury to the alveolar epithelium is thought to trigger the accumulation of fibroblasts and the deposition of ECM that characterizes IPF^1^. During the repair and remodeling process, the activated mesenchymal fibroblasts proliferate and migrate into the wound, and elevate levels of matrix proteins including collagens and fibronectin (FN), and they assume the highly synthetic myofibroblast phenotype as measured by expression of α-smooth muscle actin (α-SMA). A wide variety of mediators are involved, but TGFβ-1 is believed to be a key pro-fibrotic mediator of the fibrotic response^2^.

TGFβ, activins, inhibins, BMPs (bone morphogenic proteins), GDFs (growth differentiation factors), and GDNFs (glial-derived neurotrophic factors) belong to the TGFβ superfamily. Three isoforms of TGFβ have been identified in mammals, termed TGFβ-1, 2 and 3. In the lungs, TGFβ-1 is the most abundant isoform and is secreted by different kinds of cell types such as alveolar macrophages, neutrophils, fibroblasts, endothelial cells, and alveolar epithelial cells. Canonical TGFβ-1 signaling pathway is initiated by the active ligand binding to TβRII (TGFβ receptor II), leading to the formation of heteromeric complex of TβRI (TGFβ receptor I, also named ALK5) and TβRII on the cell membrane. Both receptors have serine/threonine kinase activity. The activated TβRI triggers intracellular signaling through phosphorylation of receptor-associated Smads (R-Smads) i.e., Smad2 and Smad3^3^. The phosphorylated R-Smad proteins form a complex with Smad4, and the heteromeric Smad complex translocates to the nucleus to regulate the transcription of target genes. Smad7, an inhibitory Smad (I-Smad), can compete with R-Smads for binding activated TβRI and inhibit R-Smad phosphorylation, thereby participating in negative feedback loops^4^,^5^. Aberrant TGFβ signaling is implicated in many human diseases including fibrosis, vascular disorders, and cancer^6^,^7^. Understanding the molecular regulatory mechanisms of TGFβ signaling, especially the molecular regulation of Smad2/Smad3, is of paramount importance for treatment of TGFβ-1-mediated human disorders.

The ubiquitin-proteasome system (UPS) is responsible for the degradation of the majority of proteins in eukaryotic cells, and plays a key role in regulating protein stability and function. Ubiquitination is the energy-dependent process in which the 8 kDa peptide ubiquitin is covalently attached to the lysine residue of a substrate protein^8^. Proteins can be poly-ubiquitinated and shuttled to the proteasome for degradation^9^,^10^. Ubiquitination-mediated proteolysis is important in a number of biological processes including signal transduction, cell cycle and gene expression^11^. It has been known that ubiquitin E3 ligases, Smurfs, and Roc1 target R-Smads for their ubiquitination and proteasomal degradation^12^,^13^.

The process of ubiquitination can be reversed by deubiquitinating enzymes (DUBs), a group of proteases that catalyze the removal of ubiquitin chains from substrate proteins^14^. Based on active site homology, DUBs can be divided into different classes including the ubiquitin-specific proteases (USPs), ubiquitin carboxyl-terminal hydrolases (UCHs), and the ovarian-tumor proteases (OTUs)^15^. There are emerging roles for DUBs as regulators of TGFβ signaling^16^,^17^,^18^,^19^,^20^ and the reversible ubiquitination of Smad proteins is a critical process that regulates the potency and duration of TGFβ signaling^21^. Ubiquitin carboxyl-terminal hydrolase-L5 (UCHL5 or UCH37) is a member of the DUBs and has been reported to interact with Smad7, and potentially reverse Smurf-mediated ubiquitination of TβRI^16^. Also, the potential of UCHL5 as a new cancer therapeutic target has been noted^22^,^23^,^24^. But the role of UCHL5 in regulation of Smad2/Smad3 and pathogenesis of pulmonary fibrosis is still unclear. Here, we demonstrate that UCHL5 de-ubiquitinates and stabilizes Smad2 and Smad3, thereby promoting TGFβ-1 signaling and contributes to the pathogenesis of pulmonary fibrosis. Targeting UCHL5 has the potential for pulmonary fibrosis treatment.

## Results

### b-AP15 attenuates TGFβ-1 signaling

**Figure 1:**
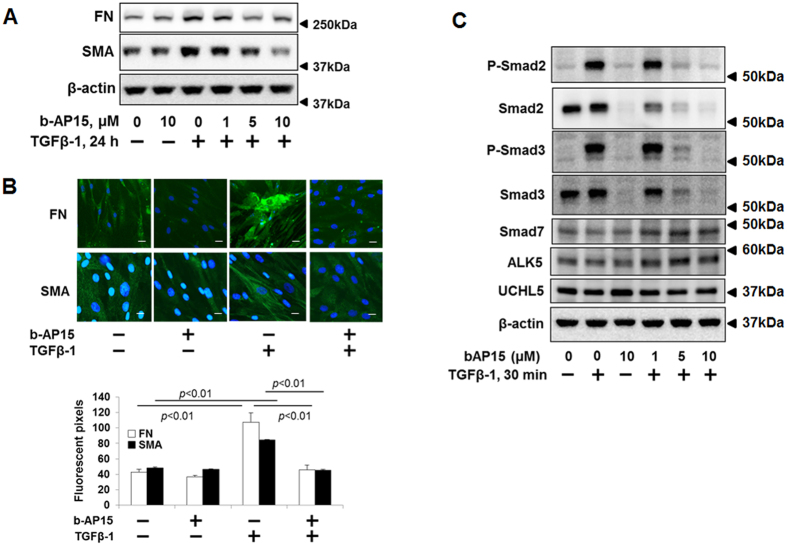
b-AP15 attenuates TGFβ-1 signaling in HLF cells. (**A**) HLF cells were pre-treated with increasing doses of b-AP15 (0, 1, 5, and 10 μM) for 1 h, and then cells were treated with TGFβ-1 (2 ng/ml) for 24 h. Cell lysates were analyzed by immunoblotting with the antibodies to FN, α-SMA, and β-actin. (**B**) HLF cells were pre-treated with DMSO and b-AP15 (50 μM) for 1 h followed by TGFβ-1 (2 ng/ml) for 24 h, and then cells were fixed with 3.7% formaldehyde. Expressions of FN and α-SMA were detected by immunostaining with antibodies to α-SMA and FN. DAPI was used for nuclei staining (blue). Scale bar, 50 μm. (**C**) HLF cells were pre-treated with increasing doses of b-AP15 (0, 1, 5, and 10 μM) for 1 h followed by TGFβ-1 (2 ng/ml) for 30 minutes. Cell lysates analyzed by immunoblotting with the antibodies to p-Smad2, p-Smad3, Smad2, Smad3, Smad7, ALK5, UCHL5, and β-actin. Western blot images were cropped to improve the conciseness of the data; samples derived from the same experiment and the blots were processed in parallel. Representative of experiments performed at least 3 independent times.

b-AP15, a nitrophenylpiperidine small molecule, inhibits the deubiquitinating activity of UCHL5 and USP14^23^. Recently, b-AP15 has been shown to have activity against multiple myeloma in a human xenograft mouse model^25^. To examine the effect of b-AP15 on TGFβ-1-induced signaling, HLF cells were pretreated with increasing doses of b-AP15 (0, 1, 5, 10 μM) for 1 h, and then treated with TGFβ-1 (2 ng/ml) for additional 24 h. It is well known that FN and α-SMA are up-regulated by TGFβ-1 and contribute to the pathogenesis of pulmonary fibrosis. First, we investigated the effect of b-AP15 on TGFβ-1-induced FN and α-SMA expression in HLF. TGFβ-1 induced FN and α-SMA expression, which were attenuated by b-AP15 in a dose-dependent manner (Fig. 1A). The data was confirmed by immunostaining (Fig. 1B). Smad2 and Smad3 are downstream signaling of TGFβ-1/TβR. Next, we examined whether b-AP15 affects Smad2/Smad3 activity. As shown in Fig. 1C, b-AP15 attenuated phosphorylation of Smad2 and Smad3 induced by TGFβ-1 treatment in a dose-dependent manner. Interestingly, total Smad2 and Smad3 levels, not Smad7, ALK5, and TβRII, were decreased by b-AP15 in both TGFβ-1 treated and untreated cells (Fig. 1C), indicating that b-AP15 may regulate Smad2 and Smad3 levels.

**Figure 2:**
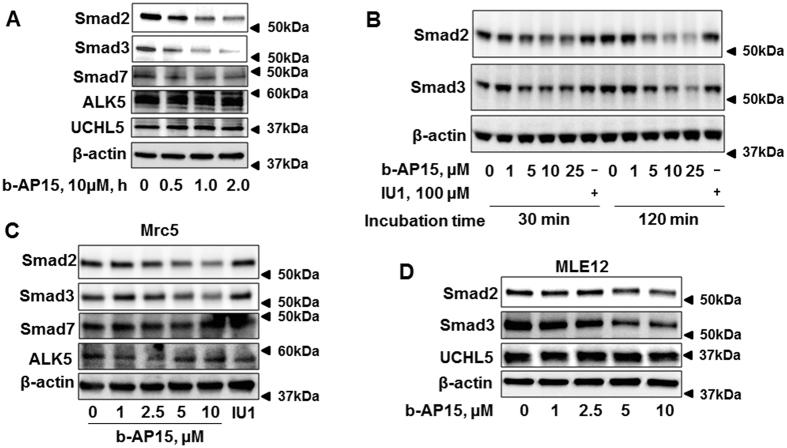
b-AP15 decreases Smad2 and Smad3 levels in HLF, Mrc5, and MLE12 cells. (**A**) HLF cells were treated with b-AP15 (10 μM) for 0–2 h. Cell lysates were analyzed by immunoblotting with the antibodies to Smad2, Smad3, Smad7, ALK5, UCHL5, and β-actin. (**B**) HLF cells were treated with different doses of b-AP15 (0, 1, 5, 10, and 25 μM) and IU1 (100 μM) for 30 min and 120 min. Cell lysates were analyzed by immunoblotting with the antibodies to Smad2, Smad3, and β-actin. (**C**) MRC5 cells were treated with different doses of b-AP15 (0, 1, 2.5, 5, and 10 μM) and IU1 (100 μM) for 1 h. Cell lysates were analyzed by immunoblotting with the antibodies to Smad2, Smad3, Smad7, ALK5, and β-actin. (**D**) MLE12 cells were treated with different doses of b-AP15 (0, 1, 2.5, 5, and 10 μM) for 1 h. Cell lysates were analyzed by immmunoblotting with the antibodies to Smad2, Smad3, UCHL5, and β-actin. Western blot images were cropped to improve the conciseness of the data; samples derived from the same experiment and the blots were processed in parallel. Representative of experiments performed at least 3 independent times.

To further investigate whether b-AP15 affects Smad2 and Smad3 expression, HLF cells were treated with b-AP15 (10 μM) for 0–2 h. Figure 2A shows that b-AP15 decreased Smad2/Smad3 in a time-dependent manner without altering the levels of Smad7, ALK5, and UCHL5. IU1 is a specific inhibitor of USP14^26^. We compared the effect of b-AP15 and IU1 on Smad2/Smad3 expression. HLF cells were treated with IU1 (100 μM) or increasing doses of b-AP15 (0, 1, 5, 10, 25 μM) for 0.5 and 2 h. Consistent with the previous findings, b-AP15 reduced Smad2/Smad3 levels in a dose-dependent manner, while IU1 had no effect on the Smad2/Smad3 (Fig. 2B). These data suggest that b-AP15 attenuates expression of Smad2/Smad3 mainly through inhibition of UCHL5 activity. The effect of b-AP15 on reduction of Smad2 and Smad3 was observed similarly in different cell types, such as Mrc5 and MLE12 (Fig. 2C,D). These data suggest that b-AP15 attenuates TGFβ-1 signaling through reduction of Smad2 and Smad3.

### b-AP15 reduces Smad2/Smad3 levels through enhancing their poly-ubiquitination and lysosomal degradation

UCHL5 is a subunit of two different complexes: INO80^27^, which performs ATP-dependent sliding of nucleosomes for transcriptional regulation and DNA repair^28^,^29^, and the 26S proteasome^30^, which performs ATP-dependent proteolysis of polyubiquitinated proteins. UCHL5 may modulate protein levels through regulation of mRNA expression and protein stability. To investigate the molecular mechanisms by which b-Ap15 reduces Smad2/Smad3 levels, first, the mRNA levels of Smad2 and Smad3 were examined by qPCR. No significant differences for Smad2 or Smad3 mRNA expression levels were detected between DMSO and b-AP15 treatment (Suppl. Fig. 1). UCHL5 disassembles lys48-linked poly-ubiquitin from the distal end of the chain^31^,^32^,^33^. Inhibition of UCHL5 by b-AP15 caused accumulation of lys48-linked poly-ubiquitination (Suppl. Fig. 2). All these data indicate that b-AP15 reduction of Smad2/Smad3 levels may be through facilitating their degradation.

**Figure 3:**
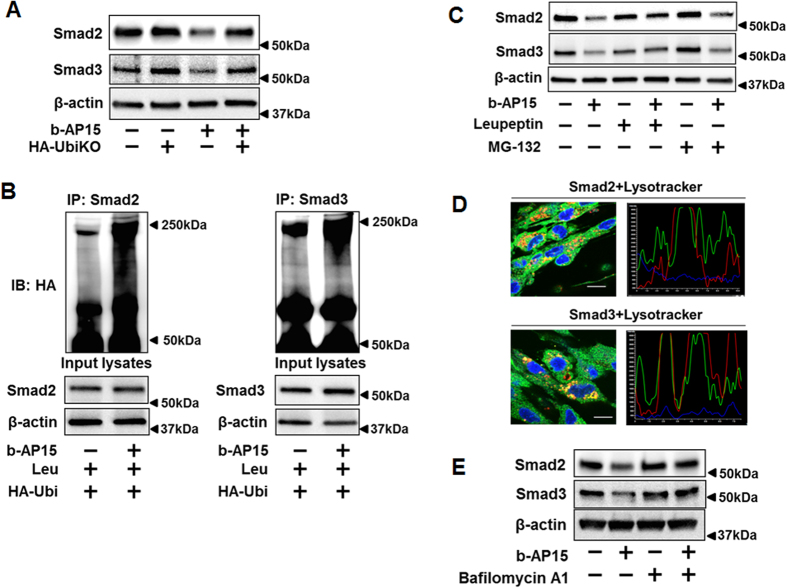
b-AP15 increases poly-ubiquitination of Smad2/Smad3 and degradation in lysosome. (**A**) HLF cells were transfected with empty vector or HA-UbiKO plasmid, and then cells were treated with b-AP15 (10 μM) for 1 h. Cell lysates were analyzed by immunoblotting with antibodies to Smad2, Smad3, and β-actin. (**B**) HLF cells were transfected with HA-tagged ubiquitin (HA-Ubi) for 48 h, and then treated with leupeptin (100 μM) for 2 h, followed DMSO and b-AP15 (10 μM) treatment for additional 1 h. Cell lysates were subjected to immunoprecipitation with Smad2 or Smad3 antibody, followed by immunoblotting with HA tag antibodies. Input cell lysates were analyzed by immunoblotting with antibodies to Smad2, Smad3, and β-actin. (**C**) HLF cells were pretreated with leupeptin (100 μM) or MG-132 (20 μM) for 2 h, then treated with b-AP15 (10 μM) for 1 h. Cell lysates were analyzed by immunoblotting with antibodies to Smad2, Smad3, and β-actin. (**D**) HLF cells were pretreated with bafilomycin A1 (10 μM) for 1 h, then cells were treated with b-AP15 (10 μM) for 1 h. Cells were fixed with 3.7% formaldehyde. Cellular location of Smad2, Smad3, and lysosome were detected by immunostaining with antibodies to Smad2 and Smad3, and lysotracker. DAPI was used for nuclei staining (blue). Scale bar, 10 μm. 94.5% of the cells with double staining show positive co-localization. (**E**) HLF cells were pretreated with bafilomycin A1 (10 μM) for 1 h, then cells were treated with b-AP15 (10 μM) for 1 h. Cell lysates were analyzed by immunoblotting with antibodies to Smad2, Smad3, and β-actin. Western blot images were cropped to improve the conciseness of the data; samples derived from the same experiment and the blots were processed in parallel. Representative of experiments performed at least 3 independent times.

To determine whether b-AP15-reduced Smad2/Smad3 is dependent on poly-ubiquitination, HLF cells were transfected with HA-UbiKO plasmid, which encodes HA tagged ubiquitin without any lysine residues and reduces poly-ubiquitination in the cells. b-AP15 reduction of Smad2 and Smad3 were attenuated in HA-UbiKO transfected cells (Fig. 3A), indicating that effect of b-AP15 on reduction of Smad2/Smad3 proteins is dependent on increases of their poly-ubiquitination. b-AP15-induced poly-ubiquitination of Smad2/Smad3 were confirmed by *in vivo* ubiquitination assay (Fig. 3B). Further to examine which pathway is involved in the Smad2/Smad3 degradation, HLF cells were pretreated with leupeptin (a lysosome inhibitor) and MG-132 (a proteasome inhibitor) for 2 h, then cells were treated with b-AP15 (10 μM) for 1 h. Leupeptin, but not MG-132, inhibited the effects of b-AP15 on reduction of Smad2/3 (Fig. 3C). Immunofluorescence staining show that Smad2 and Smad3 were co-localized with lysosome after b-AP15 treatment (Fig. 3D), indicating that Smad2/Smad3 are degraded via the lysosome pathway in presence of b-AP15. It has been shown that b-AP15 increased autophagy markers in breast cancer cells^34^. Consistent with the finding, b-AP15 treatment of HLF cells induced expression of autophagy marker, Atg12-Atg5 conjugation, not Atg7 (Suppl. Fig. 3A). Co-immunofluorescence staining revealed Smad3 was co-localized with Atg12 after b-AP15 treatment (Suppl. Fig. 3B). Further, we found that bafilomycin A1 (an autophagy inhibitor) also attenuated b-AP15-induced reduction of Smad2/Smad3 (Fig. 3E), suggesting that b-AP15-induced Smad2/Smad3 degradation is through lysosome-autophagy system.

### UCHL5 stabilizes Smad2/Smad3 and promotes TGFβ-1 signaling

**Figure 4:**
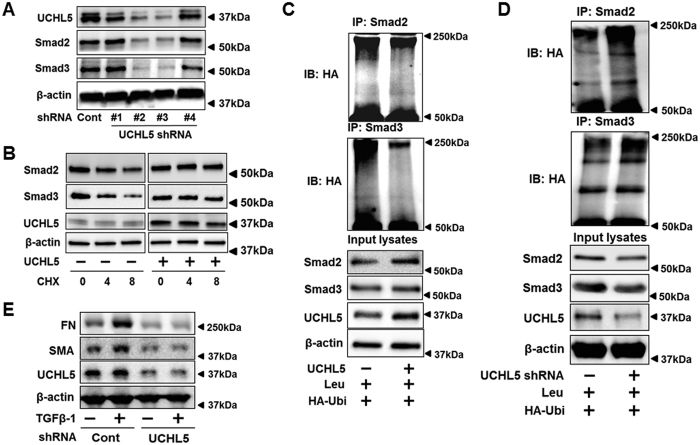
UCHL5 de-ubiquitinates and stabilizes Smad2/Smad3, and promotes TGFβ-1 signaling. (**A**) HLF cells were co-transfected with scramble shRNA and four different designed UCHL5 shRNA (#1–#4) for 3 days, and then cell lysates were analyzed by immunoblotting with the antibodies to UCHL5, Smad2, Smad3, and β-actin. (**B**) MLE12 cells transfected with empty vector plasmid or UCHL5 plasmid for 48 h, and cells were treated with CHX (20 μg/ml) for 0–8 h. Cell lysates were analyzed by immunoblotting with the antibodies to Smad2, Smad3, UCHL5, and β-actin. (**C**) HLF cells were co-transfected with HA-Ubi or HA-Ubi+UCHL5 plasmids for 48 h. Cell lysates were subjected to immunoprecipitation with Smad2 or Smad3 antibody, followed by immunoblotting with HA tag antibody. Input lysates were analyzed by immunoblotting with Smad2, Smad3, UCHL5, and β-actin. (**D**) HLF cells were transfected with scramble (cont) shRNA or UCHL5 shRNA, and then transfected with HA-Ubi plasmids for 48 h. Cell lysates were subjected to immunoprecipitation with Smad2 or Smad3 antibody, followed by immunoblotting with HA tag antibodies. Input lysates were analyzed by immunoblotting with Smad2, Smad3, UCHL5, and β-actin. (**E**) HLF cells were transfected with cont shRNA and UCHL5 shRNA for 72 h, and then cells were treated with TGFβ-1 (2 ng/ml) for 24 h. Cell lysates were analyzed by immunoblotting with the antibodies to FN, α-SMA, UCHL5, and β-actin. Western blot images were cropped to improve the conciseness of the data; samples derived from the same experiment and the blots were processed in parallel. Representative of experiments performed at least 3 independent times.

To confirm that the effect of b-AP15 on Smad2/Smad3 reduction is through inhibition of UCHL5, UCHL5 was silenced by shRNA transfection in HLF cells. UCHL5 shRNA (#2, #3, not #1, #4) diminished protein levels of Smad2/Smad3 (Fig. 4A). This was confirmed in UCHL5 shRNA lentivirus infected HLF cells (Suppl. Fig. 4). Further, we examined whether UCHL5 affects Smad2/Smad3 stability. UCHL5 was overexpressed with UCHL5 plasmid in MLE12 cells, and then cells were treated with a protein synthesis inhibitor cycloheximide (CHX) for 0–8 h. Smad2 and Smad3 had a longer half-life in UCHL5-overexpressed cells, compared to its half-life in empty vector transfected cells (Fig. 4B). Consistent with the finding from using b-AP15, UCHL5 overexpression decreased poly-ubiquitination of Smad2/Smad3 (Fig. 4C), while down-regulation of UCHL5 (~73%) by UCHL5 shRNA increased poly-ubiquitination of Smad2/Smad3 (Fig. 4D). As Smad2/Smad3 are key transcriptional factors for TGFβ-1-induced profibrotic genes, the effect of UCHL5 knockdown on expression of FN and α-SMA expression was examined. As shown in Fig. 4E, UCHL5 knockdown attenuated expression of FN and α-SMA induced by TGFβ-1, which is consistent with b-AP15 treatment in Fig. 1A. All these data show that UCHL5 stabilizes Smad2/Smad3, and promotes TGFβ-1 signaling.

### UCHL5 regulates Smad3 degradation through binding to Smad3

**Figure 5:**
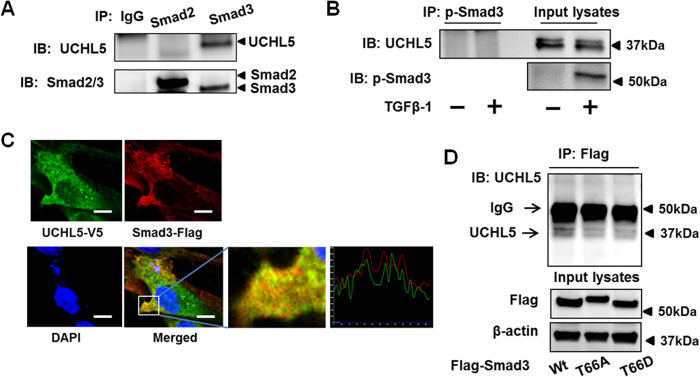
UCHL5 is associated with Smad3. (**A**) HLF cell lysates were subjected to immunoprecipitation with antibodies to IgG, Smad2, or Smad3, followed by immunoblotting with UCHL5 and Smad2/Smad3 antibodies. (**B**) HLF cells were treated with TGFβ-1 (2 ng/ml) for 30 min. Cell lysates were subjected to immunoprecipitation with an antibody to p-Smad3, followed by immunoblotting with UCHL5 antibody. Input lysates were analyzed by immunoblotting with p-Smad3 antibody. (**C**) HLF cells were co-transfected with Flag-Smad3 and V5-tagged UCHL5 (UCHL5-V5) for 48 h, and then cells were fixed with 3.7% formaldehyde. Localization of Flag-Smad3 and UCHL5-V5 were detected by co-immunostaining with antibodies to Flag tag (red) and V5 tag (green). Nuclei were stained with DAPI (blue). Scale bar, 10 μm. 92.0% of the cells with double staining show positive co-localization. (**D**) HLF cells were transfected with Flag-Smad3 wild type (Wt), Flag-Smad3T66A, and Flag-Smad3T66D plasmids for 48 h, and then cell lysates were subjected to immunoprecipitation with an antibody to Flag tag, followed by UCHL5 immunoblotting. Input lysates were analyzed by immunoblotting with Flag tag and β-actin antibodies. Western blot images were cropped to improve the conciseness of the data; samples derived from the same experiment and the blots were processed in parallel. Representative of experiments performed at least 3 independent times.

It has been reported that UCHL5 and Smad7 formed a stable complex and stabilized TβRI^16^. In HLF, we could not confirm that UCHL5 regulates TβRI stability (Figs 1C and 2A,C). Next, we examined whether UCHL5 is associated with Smad2/Smad3 using co-immunoprecipitation (co-IP). HLF cell lysates were subjected to IP with IgG, Smad2, or Smad3 antibody, followed by UCHL5, Smad2 and Smad3 immunoblotting. As shown in Fig. 5A, UCHL5 was associated with Smad3, not Smad2. To examine whether TGFβ-1 treatment enhances the association between phospho-Smad3 and UCHL5, HLF cells were treated with TGFβ-1 for 30 min, and co-IP were performed. As shown in Fig. 5B, UCHL5 was not associated with phosphorylated Smad3. Further, the co-localization of Smad3-Flag and UCHL5-V5 in HLF cells were confirmed by co-immunofluorescence staining (Fig. 5C), suggesting that UCHL5 stabilizes Smad3 by targeting unphosphorylated form of Smad3 for its de-ubiquitination. Thr66 (T66) has been shown a phosphorylation site of Smad3^35^. Here, we found that Thr66 mutants of Smad3 (Smad3T66A and Smad3T66D) have less binding efficiency to UCHL5, compared to the association between UCHL5 and Smad3 wild type (Fig. 5D), suggesting Thr66 in Smad3 is a potential binding site for UCHL5.

### UCHL5 is a potential therapeutic target for drug treatment

**Figure 6:**
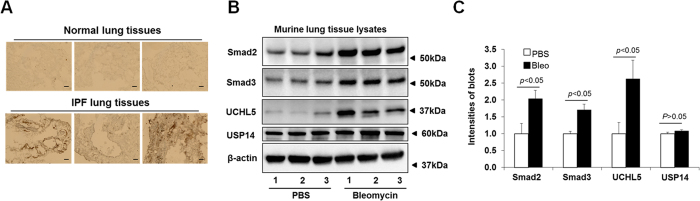
UCHL5 levels were increased in lung tissues from IPF patients and bleomycin-challenged mice. (**A**) Histological image of UCHL5 expressed in non-IPF (“Normal”) and IPF lung tissues. (**B**) C57BL/6 mice were intranasally challenged with bleomycin (0.045 U/mice) for 3 weeks, and then lung tissues were collected. Lysates from lung tissues were analyzed by immunoblotting with Smad2, Smad3, UCHL5, USP14, and β-actin antibodies. (**C**) Intensities of blots in the (**B**) were quantified by imageJ software. Western blot images were cropped to improve the conciseness of the data; samples derived from the same experiment and the blots were processed in parallel.

**Figure 7:**
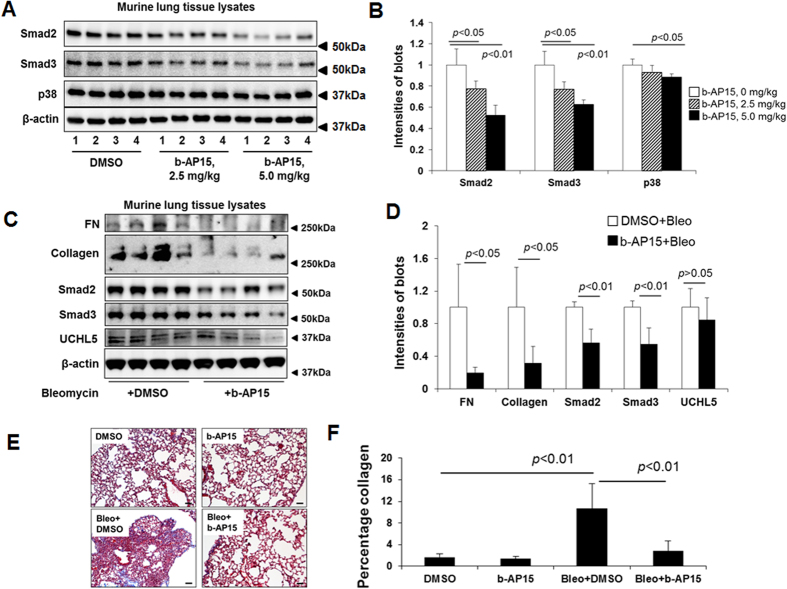
Administration of b-AP15 reduces bleomycin-induced pulmonary fibrosis in mice. (**A**) C57BL/6 mice were intraperitoneally injected with b-AP15 (2.5 mg/kg or 5.0 mg/kg, 3 × 1 every other day) for 8 days, and then lung tissues were collected. Lysates from lung tissues were analyzed by immunoblotting with antibodies to Smad2, Smad3, p38, and β-actin. (**B**) Intensities of blots in the A were quantified by imageJ software. (**C**) C57BL/6 mice were intranasally challenged with bleomycin (0.045 U/mice). Starting from day 11, mice were intraperitoneally injected with DMSO (0.25%) or b-AP15 (5.0 mg/kg in DMSO) 4 times of every other day. At day 21, lung tissues were collected and lysates from lung tissues were analyzed by immunoblotting with FN, type I collagen, Smad2, Smad3, UCHL5, and β-actin antibodies. Western blot images were cropped to improve the conciseness of the data; samples derived from the same experiment and the blots were processed in parallel. (**D**) Intensities of blots in the (**C**) were quantified by imageJ software. (**E**) The right lungs were fixed with 3.7% formaldehyde. Masson’s trichrome staining was performed to detect the collagen in the marine lung slices. (**F**) The percentages of collagen deposition in the E were analyzed by NIS-Elements software.

To further investigate the role of UCHL5 in TGFβ-1 signaling and pulmonary fibrosis, first, we examined the UCHL5 expression in lungs from IPF patients. As shown in Fig. 6A, UCHL5 is highly expressed in lungs from IPF patients, compared with its expression in lungs from non-IPF subjects. Next, we examined levels of UCHL5, Smad2 and Smad3 in murine lungs from a bleomycin-induced pulmonary fibrosis model. The UCHL5, Smad2 and Smad3 levels, not USP14, were increased in lung tissue lysates from the mice challenged by bleomycin for three weeks (Fig. 6B,C). Consistent with *in vitro* studies, b-AP15 intraperitoneal treatment (3 × 1 every other day) at doses of 2.5 mg/kg and 5.0 mg/kg could lower the levels of Smad2 and Smad3, without altering p38 MAPK in murine lungs (Fig. 7A,B). To investigate whether b-AP15 may have therapeutic potential in pulmonary fibrosis, b-AP15 (2.5 mg/kg, 4 × 1 every other day) was intraperitoneally injected beginning 11 days after bleomycin challenge. b-AP15 dramatically attenuated the levels of FN and type I collagen, and also decreased the levels of Smad2 and Smad3, not UCHL5, at 21 days after bleomycin challenge (Fig. 7C,D). Trichrome staining shows that b-AP15 decreased the deposition of collagen compared to DMSO control treatment in bleomycin-induced pulmonary fibrosis (Fig. 7E). b-AP15 may be effective therapy for pulmonary fibrosis.

## Discussion

**Figure 8:**
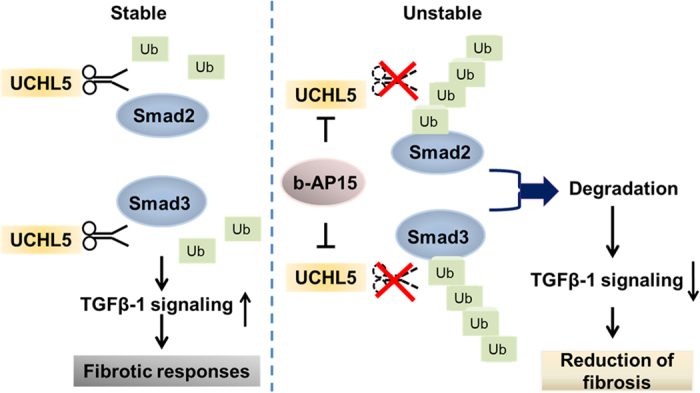
UCHL5 stabilizes Smad2/Smad3 and promotes TGFβ-1 signaling. UCHL5, which is de-ubiquitinating enzyme, catalyzes the removal of poly-ubiquitin chains from Smad2 and Smad3. Inhibition or down-regulation of UCHL5 causes Smad2/Smad3 poly-ubiquitination, promotes Smad2/Smad3 degradation, and attenuates TGFβ-1 signaling. UCHL5 is the potential target for treating fibrotic diseases.

Our data suggest a new model for TGFβ-activated signaling in pulmonary fibrosis. We have shown that UCHL5 promotes TGFβ signaling via stabilization of Smad2/Smad3 and may be a potential therapeutic target to block the differentiation of myofibroblasts and the expression of matrix in IPF. Smad2 and Smad3 are the major transcriptional factors in the TGFβ-1-mediated fibrotic response. Several ubiquitin E3 ligases have been shown to mediate ubiquitination of Smad2 and Smad3^12^,^13^. Recently, OTUB1, a DUB, was reported to reduce poly-ubiquitination of Smad2/Smad3 through inhibition of the E2 ubiquitin-conjugating enzymes. The effect is not dependent on OTUB1 catalytic activity^36^. Therefore, no DUB has been observed to de-ubiquitinate Smad2/Smad3. In the present study, we demonstrate that UCHL5 de-ubiquitinates and stabilizes Smad2 and Smad3, thereby promoting TGFβ-1 signaling. This is the first study that reveals that a de-ubiquitinating enzyme can regulate Smad2/Smad3 de-ubiquitination and stability (Fig. 8). Further, we show that UCHL5 is increased in lungs from IPF patients and bleomycin-challenged mice. This study reveals that inhibition of UCHL5 is a potential therapeutic strategy for pulmonary fibrosis treatment.

Numerous studies have illustrated different roles for DUBs including USP11, USP15, and UCHL5 in the regulation of TGFβ-1 signaling^16^,^17^,^18^,^19^,^20^. UCHL5 is of great interest since it has been reported to interact with Smad7 and potentially reverse Smurf-mediated ubiquitination of TβRI. Therefore, it stabilizes TβRI and promotes TGFβ-1 signaling in human cancers^16^. In this study, we reveal that b-AP15, an inhibitor of UCHL5, attenuates TGFβ-1 signaling through inducing ubiquitination and degradation of Smad2/Smad3. This is the first study to identify that UCHL5 plays a pro-fibrotic role in the pathogenesis of pulmonary fibrosis through stabilization of Smad2/Smad3 and enhance in TGFβ-1 signaling in HLF and a murine model of pulmonary fibrosis. This is different from the role of OTUB1, another DUB, in the regulation of Smad2/Smad3 ubiquitination^36^. OTUB1 could not directly de-ubiquitinate Smad2/Smad3, while its effect is dependent on regulating ubiquitin E2 conjugating-enzyme activity. Also, OTUB1 regulates only phosphorylated Smad2/Smad3 under TGFβ-1 treatment^36^, while UCHL5 de-ubiquitinates Smad2/Smad3 regardless of TGFβ-1 treatment. Our data show the poly-ubiquitinated Smad2/Smad3 are degraded in the lysosome-autophagy system. It has been shown that UCHL5 controls proteasome function and inhibition of UCHL5 promotes protein degradation in autophagy^34^. Here, this study shows that inhibition of UCHL5 enhances autophagy activity and induces its substrates degradation in the lysosome-autophagy system. This supports a cross-talk between proteasome- and lysosome/autophagy-mediated protein degradation. As b-AP15 may inactivate proteasome activity, this raises a concern of whether the b-AP15-reduced Smad2/3 levels are due to the dysfunction of proteasome. We show that b-AP15 increased Smad2/3 poly-ubiquitination and that UCHL5 is associated with Smad3. This suggests that there is a link between UCHL5 and Smad2/3, and it is not induced artificially to compensate the dysfunction of proteasome.

Wicks *et al.*^16^ reported that UCHL5 de-ubiquitinates and stabilizes TβRI in human cancer cells. Nevertheless, in our study, we show that b-AP15 treatment did not lower the levels of Smad7 or ALK5 (TβRI) in HLF cells. In the study by Wicks and colleagues^16^, the authors detected weak association between UCHL5 and Smad2/Smad3, while we show that UCHL5 associates with Smad3 on Thr66, not Smad2. The controversial conclusion may be due to differences in cell types. It is unclear how UCHL5 de-ubiquitinates inactivated Smad2 without protein-protein interaction in HLF cells. It is possible that a chaperone protein is involved in the process.

UCHL5 is up-regulated in most tumor tissues of ovarian cancer patients and is significantly associated with poor prognosis in human epithelial ovarian cancer^37^. Recently, UCHL5 as a new cancer therapeutic potential target has been noted^22^,^23^,^24^. We first report here that UCHL5 levels are increased in lung tissues from IPF patients and bleomycin-challenged mice. Post-treatment of b-AP15 decreases levels of Smad2, Smad3, FN and type I collagen, as well as reducing deposition of collagen in murine lung tissues from bleomycin challenged mice. Whether b-AP15 could be developed to be a potential medicine to treat IPF needs more pharmacokinetics and toxic studies. This study, at least, provides evidence that inhibition of UCHL5 lessens severity of pulmonary fibrosis in a pre-clinical animal model.

In conclusion, we demonstrate that UCHL5 de-ubiquitinates and stabilizes Smad2 and Smad3, promotes TGFβ signaling, and contributes to the pathogenesis of pulmonary fibrosis. b-AP15, an inhibitor of UCHL5, attenuates TGFβ-1 signaling and diminishes pulmonary fibrosis in a bleomycin-induced pulmonary fibrosis model. This study indicates that targeting UCHL5 may be a new potential therapeutic strategy to treat IPF. Future study will focus on the molecular regulation of UCHL5 expression in lung fibrosis.

## Materials and Methods

### Cell culture and reagents

Human primary lung fibroblast (HLF) and human lung fibroblast cell line (MRC5) cells were cultured in EMEM (Gibco) supplemented with 10% fetal bovine serum (FBS) (Hyclone) and 1% penicillin/streptomycin (Lonza). Murine lung epithelial (MLE12) cells [American Type Culture Collection (ATCC)), Manassas, VA, USA] were cultured in HITES medium complemented with 10% FBS (Hyclone). HEK293T cell line was cultured in DMEM (Gibco) supplemented with 10% FBS, 1% non-essential amino acids (MEM NEAA), 1% L-Glutamin and 1% sodium pyruvate. Cells were maintained in a 37 °C incubator in the presence of 5% CO_2_.

Immobilized protein A/G beads, FN, α-SMA, ALK5, HA tag, UCHL5, V5 tag, and control IgG antibodies were from Santa Cruz Biotechnology (Santa Cruz, California). Phospho-Smad2, phospho-Smad3, total Smad2, Smad3, and Smad7, ubiquitinK48 antibodies were purchased from Cell Signaling Technology (Danvers, MA). Bleomycin, leupeptin, cycloheximide (CHX), antibodies against Flag-tag, Type I collagen, and β-actin were from Sigma Aldrich (St. Louis, MO). Lipofectamine transfection reagent was from SignaGen Laboratories (Rockville, MD). Recombinant TGF-β1 was purchased from Invitrogen (Carlsbad, CA). b-AP15, IU1, and bafilomycin A1 were from an Cayman Chemical (Ann Arbor, MI). Proteasome inhibitor MG132 was from Calbiochem. All materials in highest grades used in the experiments are commercially available.

### Preparation of cell lysates and Western blotting

After the indicated treatments, cells were rinsed with ice-cold PBS and lysed in lysis buffer containing 20 mM Tris·HCl (pH 7.4), 150 mM NaCl, 2 mM EGTA, 5 mM β-glycerophosphate, 1 mM MgCl_2_, 1% Triton X-100, 1 mM sodium orthovanadate, 10 μg/ml protease inhibitors, 1 μg/ml aprotin, 1 μg/ml leupeptin, and 1 μg/ml pepstatin. Cell lysates were sonicated on ice for 12 s and centrifuged at 1,000 g for 10 min at 4 °C to remove cellular debris. Protein concentrations were determined with a Bio-Rad protein assay kit (Bio-Rad Laboratories, Inc) using BSA as a standard. Equal amounts of cell lysates (15 μg) were subjected to 10% SDS-PAGE, transferred to polyvinylidene difluoride membranes, membranes were blocked with 5% (wt/vol) BSA in 25 mM Tris·HCl (pH 7.4), 137 mM NaCl, and 0.1% Tween20 (TBST) for 30 min, then incubated with primary antibodies in 5% (wt/vol) BSA in TBST for at least 2 h. The membranes were washed at least 3 times with TBST at 10 min intervals and then incubated with mouse, rabbit, or goat horseradish peroxidase-conjugated secondary antibody (1:2,000) for 1 h. They were then developed with the enhanced chemiluminescence detection system according to the manufacturer’s instructions.

### Immunofluorescence staining

HLF cells were grown in glass bottom dishes until 80–90% confluence. After treatment or transfection, cells were fixed with 3.7% formaldehyde for 20 min, blocked with 1% BSA in TBST for 30 min, then immunostained with FN, α-SMA, Smad2, Smad3, Atg12, Flag tag, or V5 tag antibody followed by three washes with PBS, and incubated with the fluorescent probe-conjugated secondary antibody. Images were captured by a Nikon ECLIPSE TE 300 inverted microscope.

### Transfection of plasmids into HLF and MLE12 cells

HLF cells grown on 6-well plates or D100 plates (70–80% confluence) were transfected with Flag-tagged Smad2/Smad3 (Smad2-Flag, Smad3-Flag), HA-tagged ubiquitin (HA-Ubi), ubiquitinKO (UbiKO), V5-tagged UCHL5 (UCHL5-V5), and UCHL5 short-hairpin RNA (UCHL5 shRNA), Flag-Smad3 wild type, Flag-Smad3T66A, Flag-Smad3T66D plasmids using Polyjet^TM^ *In Vitro* DNA Transfection Reagent (SignaGen Laboratories, Inc) according to the transfection protocol, change complete media after 4 h. MLE12 cells grown on 6-well plate (70–80% confluence) were transfected with UCHL5 plasmid using Lonza electroporation transfection according to the manufacturer’s protocol. UCHL5 shRNA transfected cells were analyzed 72 h after transfection, others were analyzed 48 h after transfection. Flag-Smad3 plasmid was a gift from J. Massague (Addgene plasmid #27025), HA-UbiKO was a gift from T. Dawson (Addgene plasmid #17603).

### UCHL5 shRNA lentivirus preparation and transduction

HEK293T cell line and Lenti-X Lentivirus packaging system (Clontech Laboratories, Inc) were used for generating lentivirus according to the manufacturer’s protocol. Medium which contains lentivirus were collected and add Lenti-X concentrator in 4 °C for additional 24 h. Lentivirus was obtained after spinning down and was resuspended in PBS. The lentivirus was stored in −80 °C. For each well, 0–8 μl of UCHL5 shRNA lentivirus was mixed with 1 μl of hexadimethrine bromide (10 mg/ml). Cells were analyzed 72 h after transduction.

### RNA isolation, reverse transcription and quantitative PCR

Total RNA was isolated from cultured HLF cells using NucleoSpin RNA extraction kit (Clontech Laboratories, Inc.) according to the manufacturer’s instructions, and RNA was quantified by spectrophotometry. cDNA was prepared using the iScript cDNA synthesis kit (Bio-Rad). Quantitative PCR was performed to assess expression of Smad2/Smad3 using primers designed based on human mRNA sequences. Smad2 primers: forward 5′AGGAAGGAACAAAAGGTCCCG3′ and reverse 5′GCAAGCCACGCTAGGAAAAC3′. Smad3 primers: forward 5′CGGCCGAGCTCCCCT3′ and reverse 5′CAGGATGGACGACATGGCT3′. Real-time PCR was performed using iQ SYBR Green Supermix and the iCycler real-time PCR detection system (Bio-Rad).

### Immunoprecipitation (IP) and ubiquitin assay

Cells were washed with cold PBS and collected in cell lysis buffer. For immunoprecipitation, equal amounts of cell lysates (1 mg) were incubated with specific primary antibodies overnight at 4 °C, followed by the addition of 40 μl of protein A/G agarose beads and incubation for additional 2 h at 4 °C on a rotator. The immunoprecipitated complex was washed 3 times with phosphate-buffered saline and analyzed by immunoblotting with indicated antibodies.

For the ubiquitin assay, cells were washed and collected with cold PBS. After centrifuging at 2000 rpm for 5 min, supernatant was removed, 1 μl of ubiquitin aldehyde and 1 μl of NEM were added into the cell pellet. 50–80 μl of 2% SDS lysis buffer was added according to the cell amount. Cell lysates were then sonicated on ice for 12 sec followed by boiling at 100 °C for 10 min. Samples were diluted with TBS. The following steps were the same as normal IP.

### Bleomycin-induced murine model of pulmonary fibrosis and b-AP15 treatment

Eight-week-old C57BL/6 mice with body weight of 20–25 g (n = 32) were purchased from the Jackson Laboratory (Bar Harbor, ME). Bleomycin (0.045 U) was administered by intranasal injection^38^. To examine the therapeutic effect of b-AP15, on day 11 after bleomycin injury or saline controls, b-AP15 (5 mg/kg), or the same volume of DMSO, was given intraperitoneally every other day until the mice were sacrificed on day 21. The right lungs were inflated and fixed with 4% paraformaldehyde (USB Corporation, Cleveland, OH), and then were ligated at the bronchi, excised, and fixed by immersion in 4% paraformaldehyde for at least 24 h before paraffin embedding and sectioning. Routine hematoxylin and eosin (H&E) and Masson’s trichrome staining was performed by the University of Pittsburgh histology facility. Partial right lungs were homogenized in cell lysis buffer. Protein levels were analyzed by Western blotting using indicated antibodies. All animal procedures in this study were performed in adherence with the National Institute of Health Guidelines on the use of Laboratory Animals and have been approved by the Institutional Animal Care and Use Committee of the University of Pittsburgh.

### Statistical analysis

All results were subjected to statistical analysis using Microsoft Excel or ANOVA, and, wherever appropriate, the data were analyzed by Student’s t-test and expressed as means ± SD. Data were collected from at least three independent experiments, and p < 0.05 was considered significant.

## Additional Information

**How to cite this article**: Nan, L. *et al.* Ubiquitin carboxyl-terminal hydrolase-L5 promotes TGFβ-1 signaling by de-ubiquitinating and stabilizing Smad2/Smad3 in pulmonary fibrosis. *Sci. Rep.* **6**, 33116; doi: 10.1038/srep33116 (2016).

## Supplementary Material

Supplementary Information
