# Supplementary material for: Ubiquitin carboxyl-terminal hydrolase-L5 promotes TGFβ-1 signaling by de-ubiquitinating and stabilizing Smad2/Smad3 in pulmonary fibrosis
Source: Sci Rep. 2016 Sep 8;6:33116. doi: 10.1038/srep33116 (PMC5015047; doi:10.1038/srep33116)
Supplement: Supplementary Information [file srep33116-s1.pdf]

Supplemental support data

Ubiquitin carboxyl-terminal hydrolase-L5 promotes TGFβ-1 signaling by de-ubiquitinating and stabilizing Smad2/Smad3 in pulmonary fibrosis

Ling Nan [1], Anastasia M Jacko [2], Jiangning Tan [2], Dan Wang [1], Jing Zhao [2], Daniel J Kass [2], Haichun Ma [1†], Yutong Zhao [2#†]

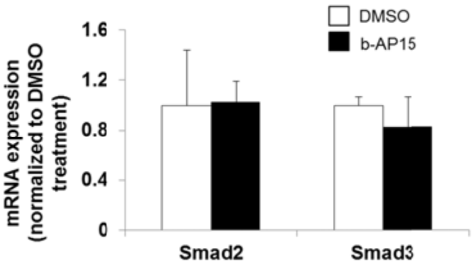

Suppl. Fig. 1. b-AP15 has no effect on mRNA levels of Smad2 and Smad3. HLF cells were treated with b-AP15 (10 μM) for 1 h. Total RNA was isolated and cDNA was synthesized by reverse transcription. Quantitative PCR was performed to assess mRNA expression of Smad2 and Smad3.

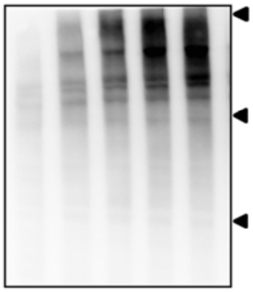

Suppl. Fig. 2. b-AP15 causes accumulation of poly-ubiquitination in HLF cells. HLF cells were treated with different doses of b-AP15 (0, 1, 5, 10, 25 μM) for 1 h and then cell lysates were analyzed by immunoblotting with an ubiquitinK48 antibody. Western blot images were cropped to improve the conciseness of the data; samples derived from the same experiment and the blots were processed in parallel. Representative of experiments performed at least 3 independent times.

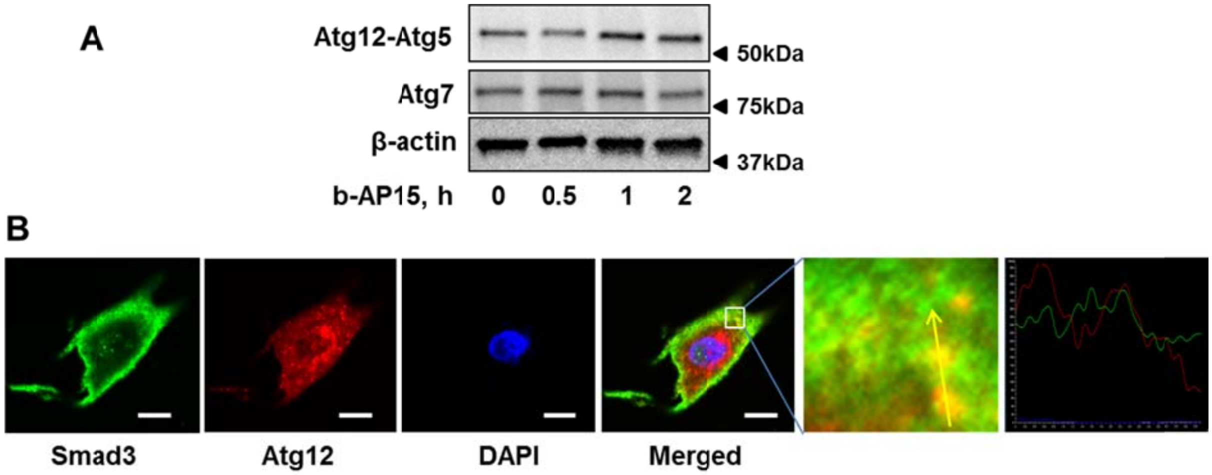

Suppl. Fig. 3. b-AP15 increases Atg12-Atg5 conjugation and co-localization of Smad3 and Atg12. A. Mrc5 cells were treated with b-AP15 (5 µM) for 0-2 h. Cell lysates were analyzed by Atg12, Atg7, and β-actin antibodies. Western blot images were cropped to improve the conciseness of the data; samples derived from the same experiment and the blots were processed in parallel. Representative of experiments performed at least 3 independent times. **B.** HLF cells were treated with bafilomycin A1 (10 µM, 1 h) prior to b-AP15 (5 µM) for 1 h. The localization of Smad3 and Atg12 were detected by immunofluorescence staining. Smad3, green; Atg12, red; nuclei, blue. Scale bar, 10 µm. Co-localization was analyzed by NIS-Elements software. 94% of the cells with double staining show positive co-localization.

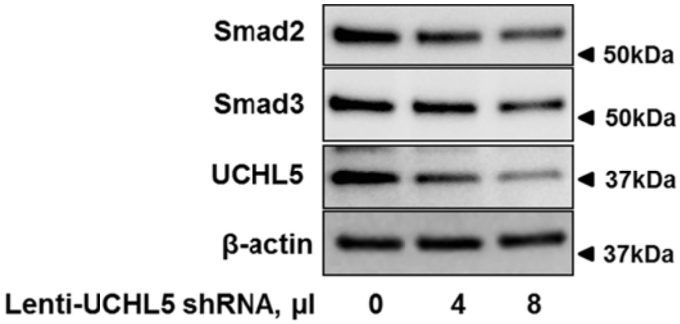

Suppl. Fig. 4. UCHL5 shRNA reduces Smad2/Smad3 levels. HLF cells were infected with different doses of lentivirus vector containing UCHL5 shRNA (0, 4, and 8 μl) and incubated for 72 h. Cell lysates were analyzed by Smad2, Smad3, UCHL5, and β-actin antibodies. Western blot images were cropped to improve the conciseness of the data; samples derived from the same experiment and the blots were processed in parallel. Representative of experiments performed at least 3 independent times.

Fig. 1A

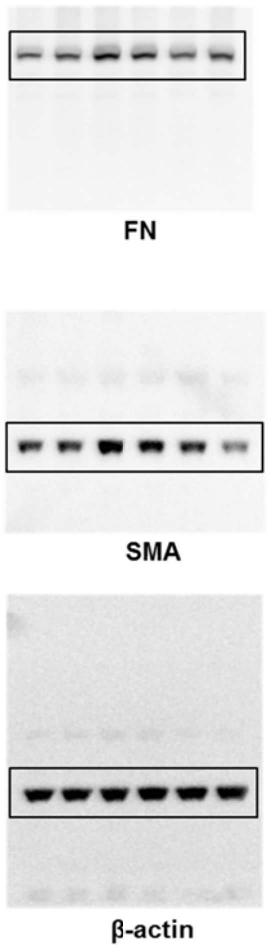
FN
SMA
β-actin

Fig. 1C

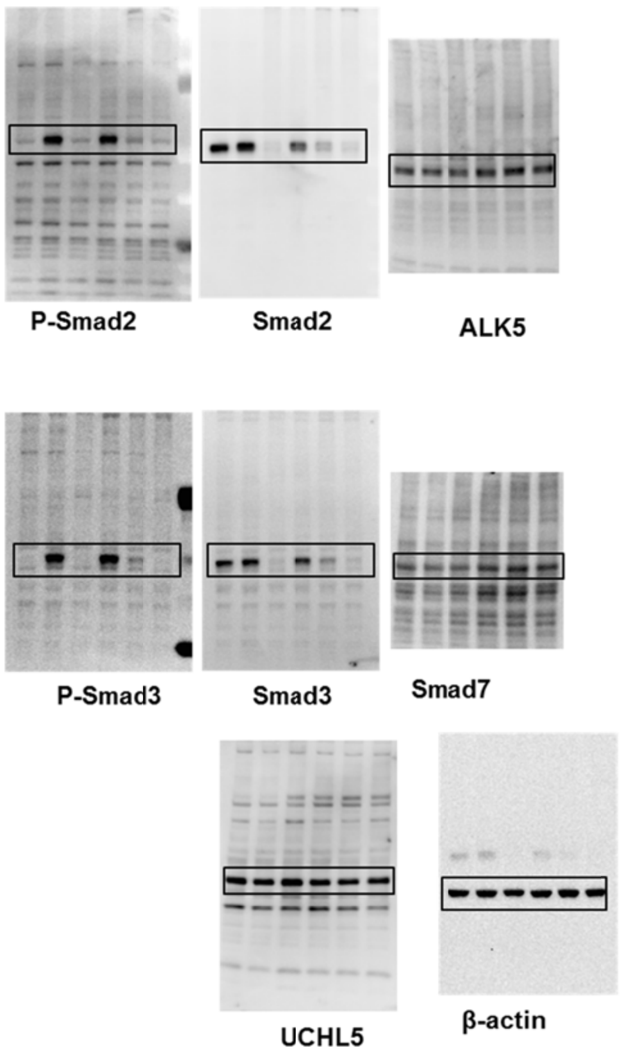
P-Smad2
Smad2
ALK5
P-Smad3
Smad3
Smad7
UCHL5
β-actin

Fig. 2A

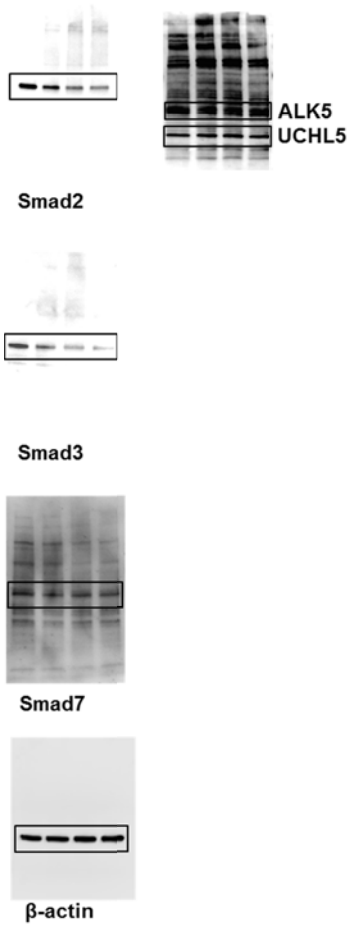

Fig. 2B

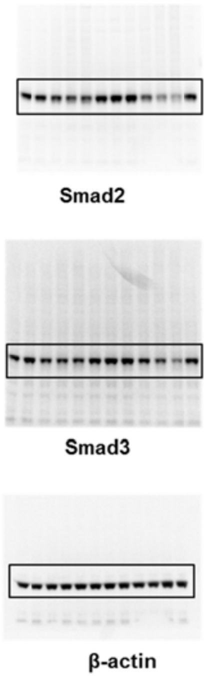

Fig. 2C

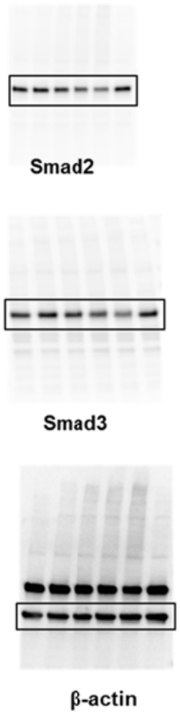

Fig. 2D

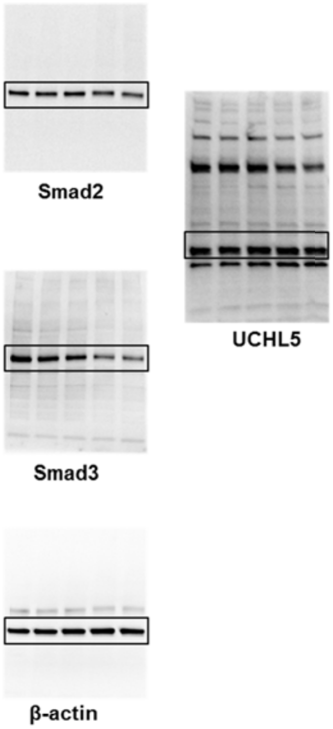

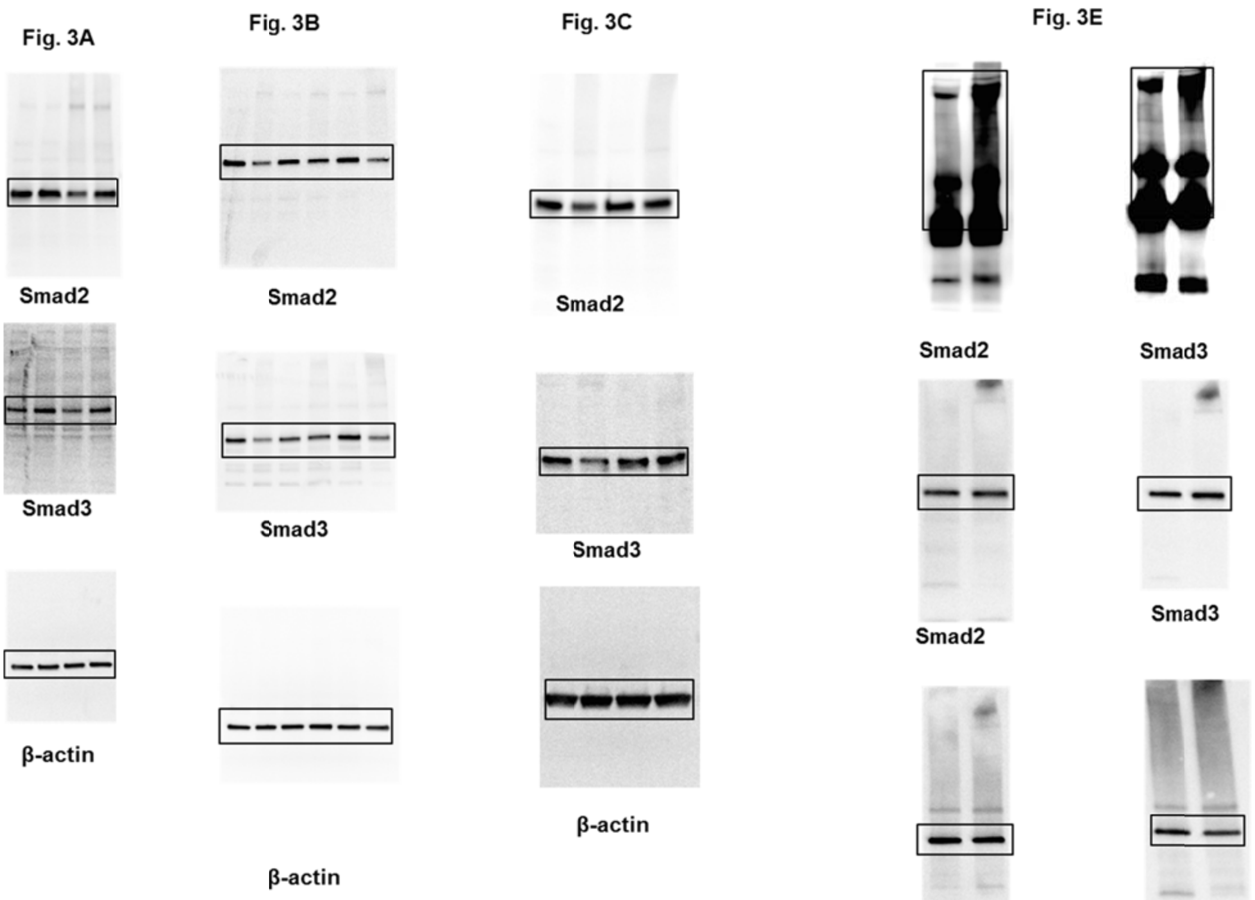
Fig. 3A
Smad2
Smad3
β-actin
Fig. 3B
Smad2
Smad3
β-actin
Fig. 3C
Smad2
Smad3
β-actin
Fig. 3E
Smad2
Smad3
Smad2
Smad3
β-actin
β-actin

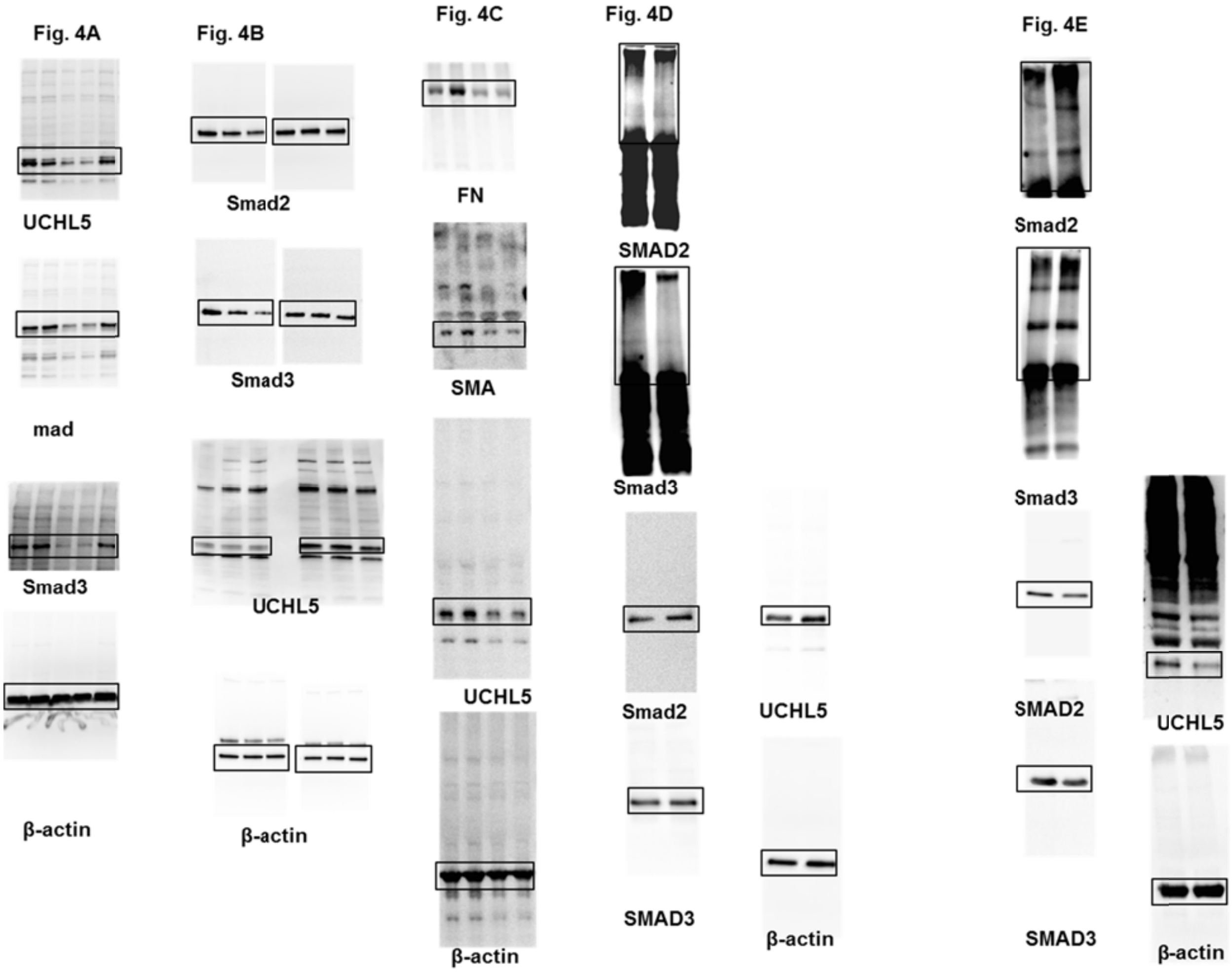

Fig. 4A
UCHL5
mad
Smad3
β-actin
Fig. 4B
Smad2
Smad3
UCHL5
β-actin
Fig. 4C
FN
SMA
UCHL5
β-actin
Fig. 4D
SMAD2
Smad3
Smad2
UCHL5
SMAD3
β-actin
Fig. 4E
Smad2
Smad3
SMAD2
UCHL5
SMAD3
β-actin

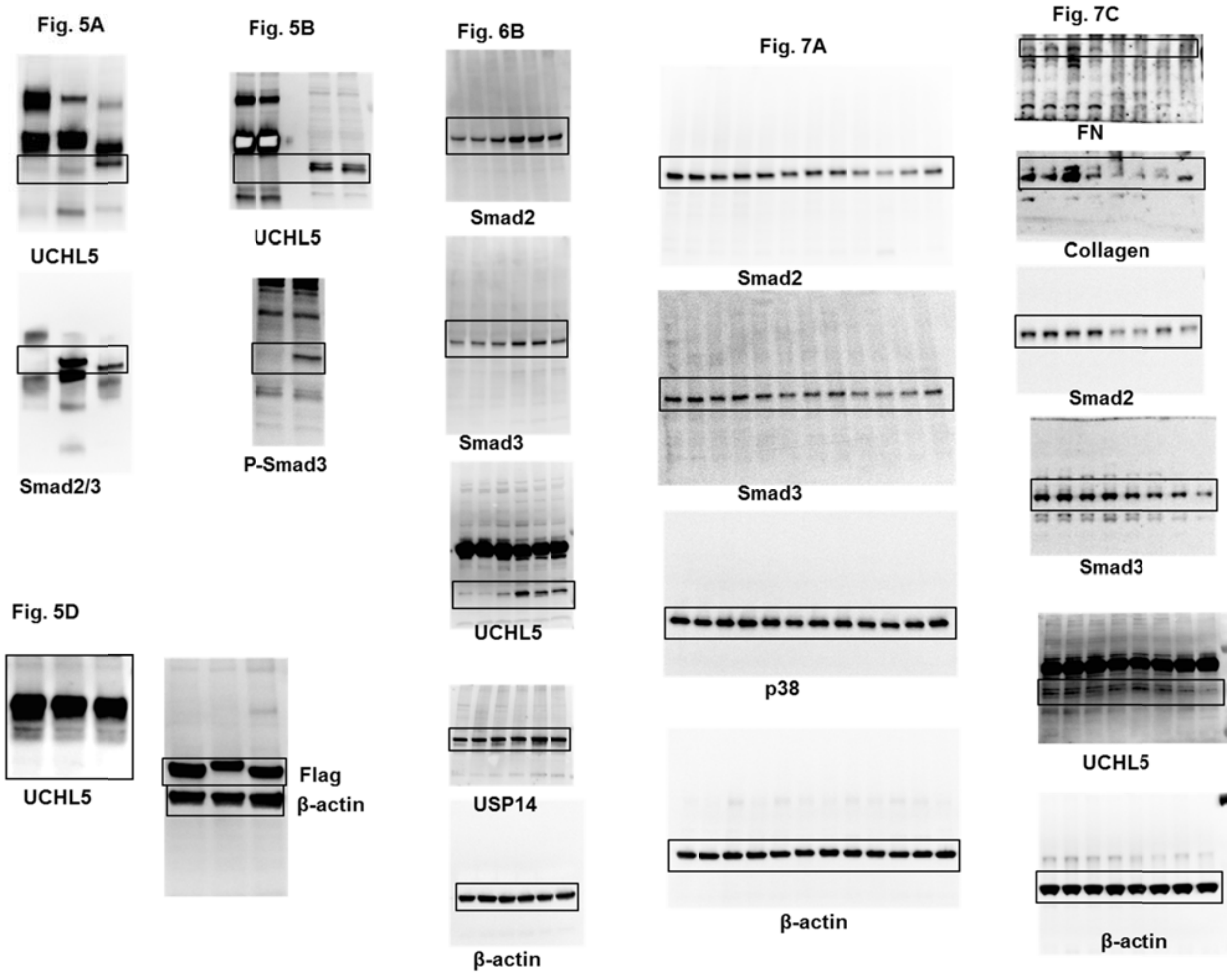
Fig. 5A
UCHL5
Smad2/3
Fig. 5B
UCHL5
P-Smad3
Fig. 5D
UCHL5
Flag
β-actin
Fig. 6B
Smad2
Smad3
UCHL5
USP14
β-actin
Fig. 7A
Smad2
Smad3
p38
β-actin
Fig. 7C
FN
Collagen
Smad2
Smad3
UCHL5
β-actin
